# Metal Coated Colloidosomes as Carriers for an Antibiotic

**DOI:** 10.3389/fchem.2018.00196

**Published:** 2018-06-01

**Authors:** Qian Sun, Ziyan Zhao, Elizabeth A. H. Hall, Alexander F. Routh

**Affiliations:** ^1^Department of Chemical Engineering and Biotechnology, BP Institute, University of Cambridge, Cambridge, United Kingdom; ^2^Department of Chemical Engineering and Biotechnology, University of Cambridge, Cambridge, United Kingdom

**Keywords:** encapsulating, impermeable, silver shells, gold shells, *E. coli*

## Abstract

Colloidosomes are polymer shell microcapsules. They are stable and easy to prepare and have been used to encapsulate drugs for release at specific areas in the body. Traditional polymer shell capsules cannot totally seal drugs, since they are porous, and small molecules diffuse through the polymer shell. In this paper, we report a method for encapsulating an antibiotic kanamycin using gold or silver coated colloidosomes. The colloidosomes are impermeable and can be triggered using ultrasound. To investigate the application of the capsules in a biological system, *Escherichia Coli* (*E. coli*) was chosen as a model organism. After triggering, the released antibiotic, as well as the metal shell fragments, kill *E. coli*. Both the silver and gold shells colloidosomes are toxic to this bacterial system and the gold coated colloidosomes can load a higher concentration of kanamycin.

## Introduction

Small molecular weight drugs are widely used in pharmaceutical applications. In order to deliver the encapsulant to targeted areas, drug delivery vehicles are developed and have attracted considerable interest. Many systems can be used as drug delivery vehicles, including micro-emulsions (Osborne et al., 1991; Kogan and Garti, 2006; Lawrence and Rees, 2012), nanotubes (Liu et al., 2008), metal organic frameworks (Horcajada et al., 2006, 2010), nanogels (Zha et al., 2011), polymers (Pillai and Panchagnula, 2001; Schmaljohann, 2006; Cho et al., 2008), and capsules (Delcea et al., 2001; Wang et al., 2008; Yang et al., 2008).

Optimally, the drug delivery vehicle should display a sufficient circulation lifetime to allow a successful delivery to the targeted region. Furthermore, for toxic drugs, encapsulation in drug carriers reduces the harmful side effects (Chertok et al., 2008; Ho et al., 2009). Among drug delivery vehicles, microcapsules have attracted considerable interest and development, since they are stable and easy to prepare. In addition, one can easily load the drug and achieve release at targeted areas (Qiu and Park, 2001; Zhao et al., 2007; Hu et al., 2008; Broaders et al., 2011). However, traditional polymer shell capsules cannot totally seal small molecule encapsulated drugs, since they are porous and diffusion occurs through the polymer shells (Shi and Caruso, 2001; Yow and Routh, 2006; Keen et al., 2014). One solution is to place a second impermeable layer around the microcapsules in order to enhance the seal ability and stability (Mandal et al., 2010; Sander and Studart, 2011; Hitchcock et al., 2015; Gao et al., 2016).

**Figure 1:**
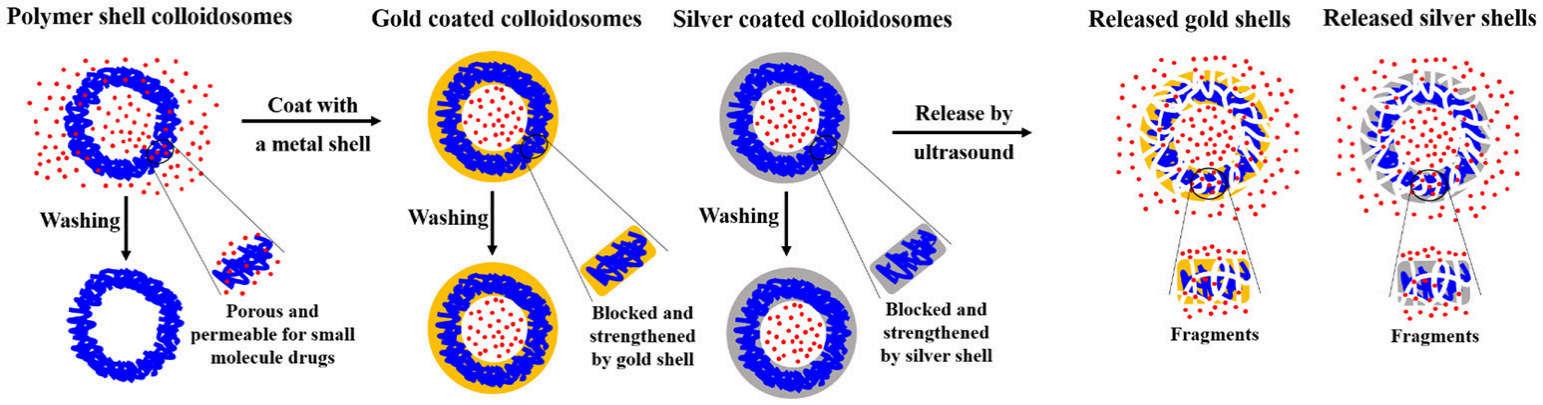
Schematic representation of microcapsule encapsulation and release of small molecules. Blue lines represent latex particles, the red represents small molecule drugs, the orange represents the gold particles, and gray the silver particles.

In our previous studies, we made impermeable metal coated colloidosomes, which were polymer shell capsules coated with either gold or silver (Sun and Routh, 2016; Sun et al., 2017a,b). Figure 1 shows a schematic representation of the encapsulation method and the release of small molecules. As shown, the polymer shells lose small molecules after washing. However, the metal shell blocks and strengthens the porous polymer. To release the encapsulated materials, ultrasound was used, breaking most of the capsules.

In this paper, to investigate the application of the metal coated colloidosomes in a biological system, *Escherichia Coli* (*E. coli*) bacteria were mixed with colloidosomes containing the antibiotic kanamycin. *E. coli* have been studied extensively and are an ideal model organism. Kanamycin is an aminoglycoside antibiotic that induces its bactericidal effect by introducing errors in protein synthesis through tRNA mismatch upon binding to the bacterial ribosome (Feldman et al., 2010). Here, we report the drug loading method using gold and silver coated colloidosomes. We then use ultrasound to break the capsules and release the drugs. After triggering, the released antibiotic, and broken shell fragments both kill *E. coli*.

## Materials and methods

### Materials

The base latex particles are poly (methyl methacrylate-co-butyl acrylate) with a diameter of 153 nm. They were synthesized via emulsion polymerization as reported elsewhere (Keen et al., 2014). The glass transition temperature of the latex was found using differential scanning calorimetry to be 35°C.

The deionized water in all experiments had a resistivity of 18.2 MΩ∙cm and was produced by a Pure Lab Ultra apparatus. Sodium dodecyl sulfate (SDS, Fisher Scientific), buffer solution pH 10.0 (Sigma-Aldrich), 4,4′-dithiodibutyric acid (DDA, 95%, Sigma-Aldrich), and kanamycin monosulphate (Sigma-Aldrich) were all used as received without purification. The vortex mixer was a TopMix FB15024 (Fisher Scientific).

For cell viability studies, pET-24a (+) plasmid with a kanamycin resistant site (Novagen) was transferred into *E. Coli* BL21 (DE3) competent cells (Novagen). The cells were grown in kanamycin containing Luria Bertani (LB) Media. The LB media was prepared with 10 g/L tryptone (mirobiologically tested, Sigma-Aldrich), 5 g/L yeast extract (for use in microbial growth medium, Sigma-Aldrich), 10 g/L NaCl (Sigma-Aldrich), and 1.5% (w/v) Agar powder (ThermoFisher Scientific).

### Drug encapsulation using gold coated colloidosomes

**Figure 2:**
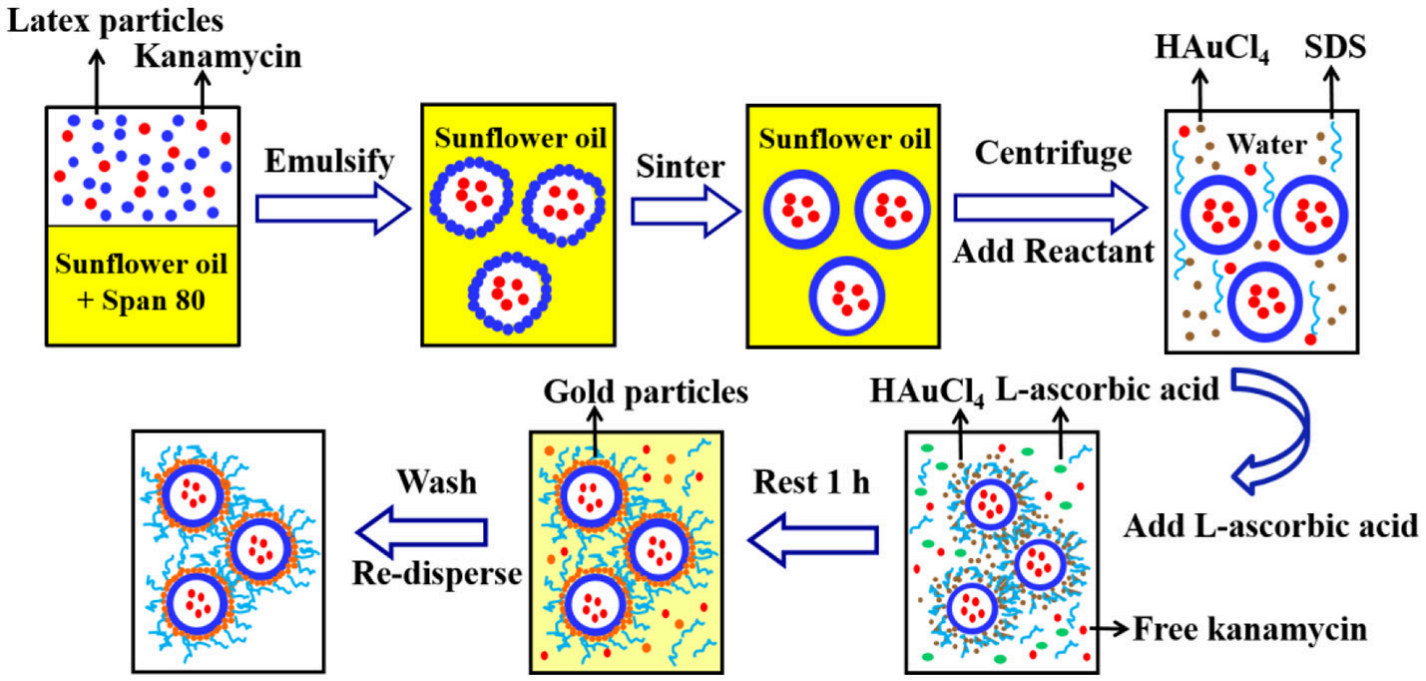
A typical method for fabrication of the gold coated colloidosomes loading kanamycin monosulphate.

Figure 2 shows the fabrication method for gold coated colloidosomes loading kanamycin monosulphate. A Silverson high shear mixer (model SL2) was used to mix 4 mL Span 80 with 200 mL sunflower oil in a 400 mL beaker. The latex particle suspension (11.2 wt% in pH 10 buffer solution) was mixed with 12.5 mg/mL kanamycin monosulphate, to get a mixture, which contained 5.6 wt% latex particles and 6.25 mg/mL kanamycin monosulphate in buffer solution. 2 mL of this mixture was then added into the sunflower oil. After emulsification, the mixture was heated in a water bath at 50 ± 0.5°C. This allows the latex particles to merge into a smooth shell.

After sintering, 20mL of the emulsion mixture was centrifuged at 2,500 rpm for 10 min at 20°C. The oil was removed via pipetting and 20 mL of 1 wt % aqueous solution of HAuCl_4_ and 2mL of 1 wt% aqueous SDS solution were added and the microcapsules redispersed in the aqueous phase using a vortex mixer. Then 2mL L-ascorbic acid solution (15 wt% in water) was added to the tube and rested for 1 h to allow the gold forming reaction. After the reduction reaction, the mixture was centrifuged at 1,500 rpm for 2 min at 20°C to recover the sediment and the supernatant was removed via pipetting. The resulting gold coated colloidosomes, loaded with kanamycin, were washed, and redispersed, using a 0.1 wt% SDS solution.

The SDS surfactant solution affects the cell viability. However, without the surfactant the capsules are heavily aggregated in water. Consequently we used 4,4′-dithiodibutyric acid (DDA) to modify the capsule surfaces, which allows the metal shell capsules to disperse in water. A known mass of gold coated colloidosomes were dispersed in 20mL of 0.5 wt% 4,4′-dithiodibutyric acid (DDA) in ethanol using the vortex. The mixture was then mixed by a magnetic stirrer for 48 h at room temperature. After the reaction, the mixture was centrifuged at 1,500 rpm for 2 min. The supernatant was removed and the modified gold shell capsules were washed and redispersed using ultra-pure water.

### Drug encapsulation using silver coated colloidosomes

The method of silver shell fabrication is similar to that for the gold shell capsules. For silver shells, 24mL AgNO_3_ solution (0.1 wt% in water) and 2mL SDS (1 wt% in water) were added in each tube. Then 2mL L-ascorbic acid solution (15 wt% in water) was added and rested for 1 h allowing the silver forming reaction.

### Release by ultrasonic treatment

Remote activation of microcapsules was conducted using an ultrasonic probe operating at a frequency of 23 kHz and 50 W. The suspension of microcapsules was subjected to ultrasound sonication, performed using an ultrasonic processor (Sanyo soniprep 150). The probe was placed into a 5mL capsule suspension in a 50mL plastic tube. An ice bath was applied to ensure that the temperature change of the capsule suspension was less than 5°C.

### Cell viability test

The kanamycin resistant *E. coli* BL21 (DE3) cells were grown for 16 h in 10mL LB medium supplemented with 50μg/mL kanamycin. 100μL of the overnight culture were then transferred to 50mL falcon tubes containing the same LB medium for inoculation. The growth of cells was monitored by measuring the optical density at 600 nm (OD_600_) of the cell culture using a UV-visible spectrophotometer (Infinite M200 Tecan). The treatments were added to the cells, when the culture OD_600_ was approximately 0.7. For blank kanamycin measurements, 1mL of kanamycin solution at various concentrations was added to the cells to obtain final concentrations between 50 and 5,000μg/mL. For metal shell capsules, blank silver/gold shells and silver/gold shells loading kanamycin with intact shells were added to the cell culture, achieving a final concentration in the range of 7.1 × 10^5^ to 3.5 × 10^8^ capsules/mL. A hemocytometer (a counting chamber) was used for determining the number of microcapsules per unit volume of a suspension. For drug release experiments, the same concentrations of ultrasound-broken capsules were added to the cell culture. Treated cells were then grown for 24 h. All cultures were shaken at 37°C, 225 rpm in a multitron shaker (Infors HT). The resulting cell cultures were then diluted in LB media and spread evenly on a 90 mm LB Agar plate supplemented with 50μg/mL kanamycin, to result in 30-300 CFU per agar plate. The plates were then grown at 37°C in an incubator (Heraeus Instruments) for 16 h before the CFU for each plate was counted.

### Sample characterizations

The colloidosomes were imaged by scanning electron microscopy (SEM), using a Zeiss X-beam FIB SEM at an accelerating voltage of 5.0 kV. A drop of colloidosome suspension was air-dried on a stainless steel SEM stub overnight and the samples were imaged without any treatment.

The elemental analysis of colloidosome samples was detected by Energy-dispersive X-ray spectroscopy (EDX). The electron beam excitation used is from a Zeiss X-beam FIB scanning electron microscope with an accelerating voltage of 10.0 kV.

## Results and discussion

### Drug encapsulation using metal coated colloidosomes

**Figure 3:**
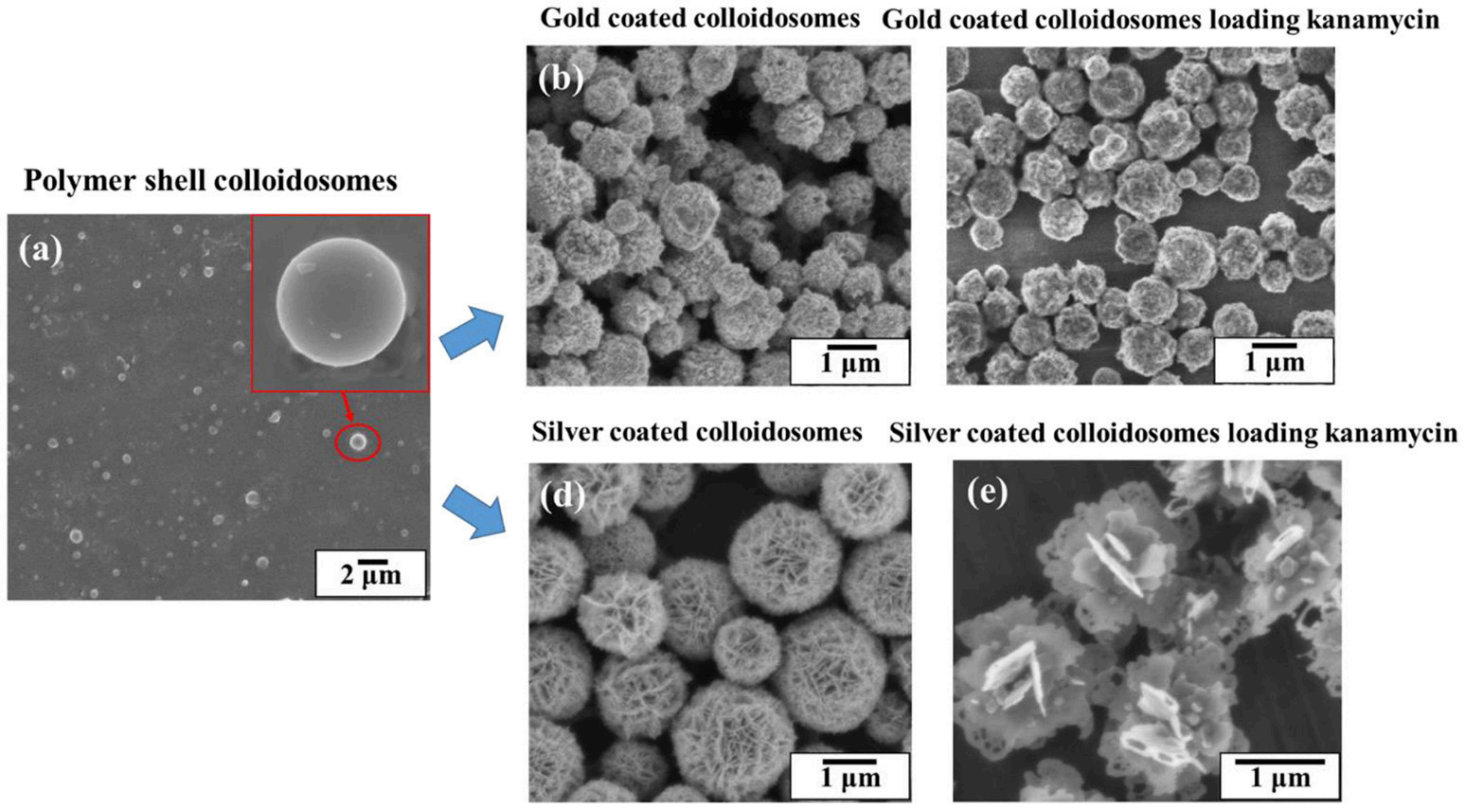
SEM images of **(a)** a polymer shell colloidosome, **(b)** gold coated colloidosomes, **(c)** gold coated colloidosomes loading kanamycin, **(d)** silver coated colloidosomes, and **(e)** silver coated colloidosomes loading kanamycin.

Figure 3 shows SEM images of (a) a polymer shell colloidosome, (b) gold coated colloidosomes, (c) gold coated colloidosomes loading kanamycin, (d) silver coated colloidosomes, and (e) silver coated colloidosomes loading kanamycin. In our previous studies, we made smooth polymer shell capsules, with a diameter of between 0.7 and 2μm and the gold or silver shell was then placed on the outside. The diameter of the gold coated colloidosomes are between 0.8 and 2.1μm, whilst the silver capsules are between 0.9 and 3.2μm. We used a fluorescent dye as a model to test the loading efficiency of the metal shells. For gold shells, the loading efficiency is around 15.8, and 22.7% for silver shells. (Sun and Routh, 2016; Sun et al., 2017a,b) Once made the metal coated colloidosomes are stable for at least many months. In this paper we report on the use of the metal coated colloidosomes for delivery of the antibiotic kanamycin into a biological system. There are some interested morphological changes to the capsules upon encapsulation of the antibiotic and we report on these first.

**Figure 4:**
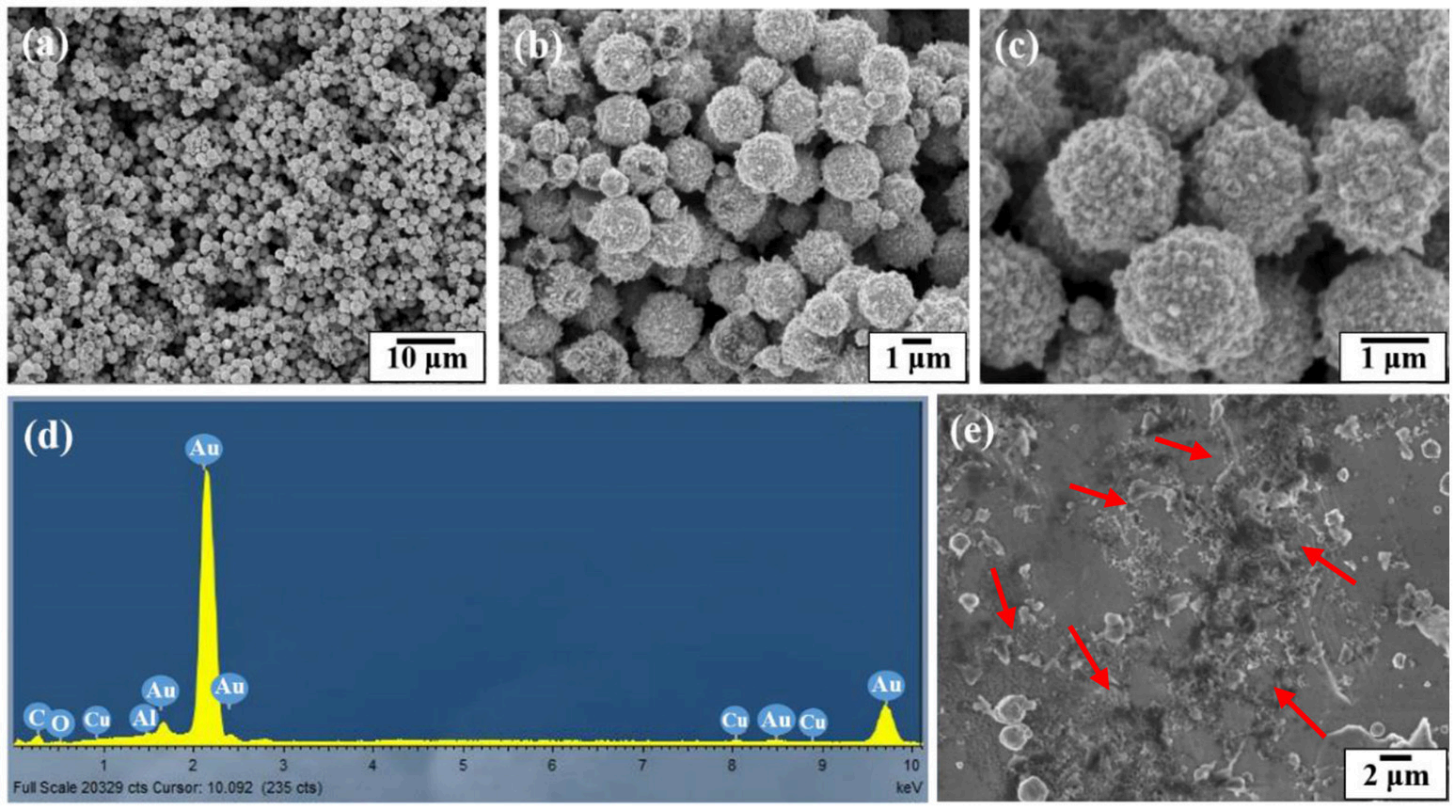
SEM and EDX images of the gold coated colloidosomes which contained antibiotic kanamycin. **(a–c)** different magnification SEM images of gold coated colloidosomes loading kanamycin, **(d)** EDX image of gold coated colloidosomes loading kanamycin, and **(e)** SEM image of gold coated colloidosomes loading kanamycin after 480 s sonication.

Figure 4 shows the SEM and EDX images of the gold coated colloidosomes containing kanamycin. It can be seen that the polymer shells were fully covered by gold particles. After loading kanamycin, the morphology of the gold shells does not significantly change, and the capsules remain spherical. Figure 4d shows the corresponding EDX image which has high gold peaks, suggesting that the particles surrounding the surface of the colloidosomes are gold. There are also carbon and oxygen peaks, arising from the polymer shell. The aluminum and copper peaks were caused by the SEM stub. To release the encapsulated drug, ultrasound was used to trigger the capsules. As shown in Figure 4e, after 480 s sonication, only a few colloidosomes survived and there were a large number of small pieces of broken shell.

**Figure 5:**
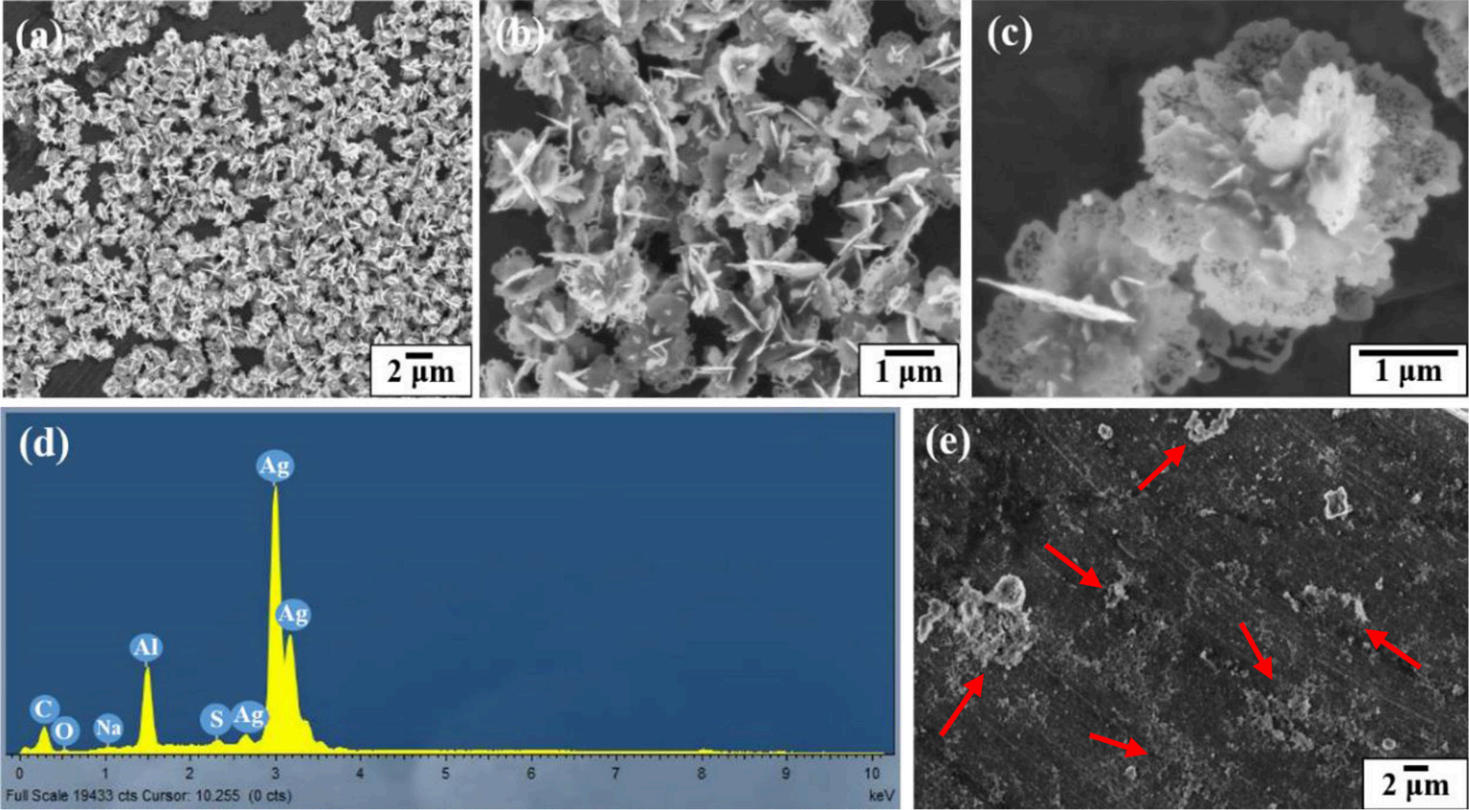
SEM and EDX images of the silver coated colloidosomes which contained antibiotic kanamycin. **(a–c)** different magnification SEM images of silver coated colloidosomes loading kanamycin, **(d)** EDX image of silver coated colloidosomes loading kanamycin, and **(e)** SEM image of silver coated colloidosomes loading kanamycin after 240 s sonication.

Figure 5 shows the SEM and EDX images of the silver coated colloidosomes which contained kanamycin. As can be seen, the polymer shells were fully covered by lamellar silver particles. After loading kanamycin, the morphology of the silver shells change from the water core capsules shown in Figure 3d. The lamellar silver particles, surrounding the surface of the polymer shells, were thinner and some silver sheets became hollow at the edge. In the silver forming process, the kanamycin may affect the silver ions converting into metallic silver. This is likely because kanamycin might associate with the silver precursor, and prevent it from forming shell shaped capsules. (Xu et al., 2015).

Figure 5d shows the corresponding EDX image which has high silver peaks. The sodium and sulfur peaks were caused by the SDS surfactant. As shown in Figure 5e, after 240 s sonication, most of the silver coated colloidosomes are broken into fragments.

**Figure 6:**
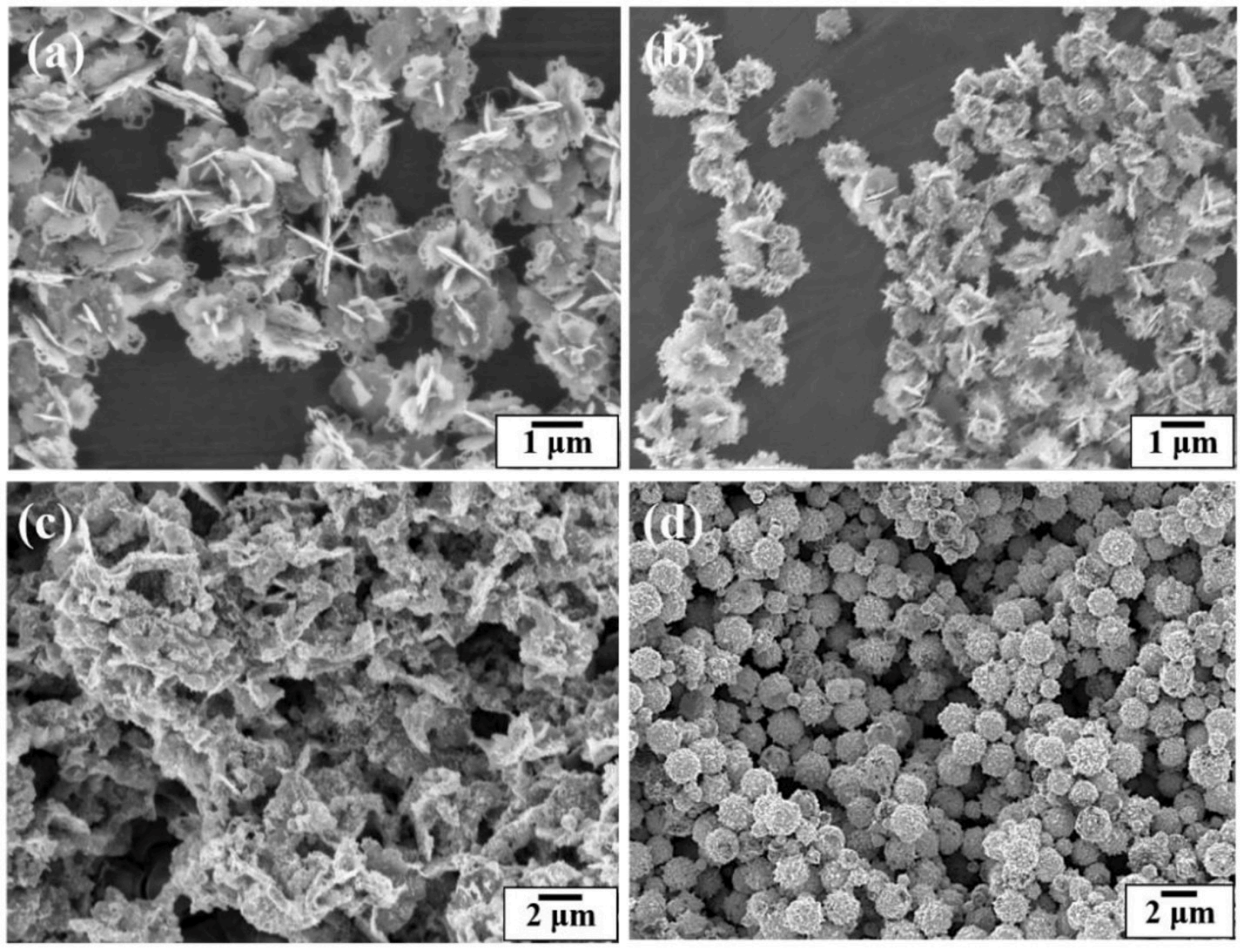
SEM images of the silver coated colloidosomes loading various concentrations of kanamycin **(a)** 12.5 mg/mL, **(b)** 25.0 mg/mL, and **(c)** 50.0 mg/mL kanamycin. **(d)** SEM image of the gold coated colloidosomes loading 50.0 mg/mL of kanamycin.

Figure 6 shows SEM images of the silver coated colloidosomes encapsulating varying amounts of kanamycin. When the original kanamycin concentration was increased to 25.0 mg/mL, the silver particles became thinner. When the concentration increased to 50.0 mg/mL, there was no silver shell, just separate silver sheets. However, for gold shells, there was no effect of kanamycin concentration as shown in Figure 6d. For comparison, SEM images of gold and silver shell colloidosomes made without kanamycin are shown in Figures 3b,d.

### Cell viability studies

**Figure 7:**
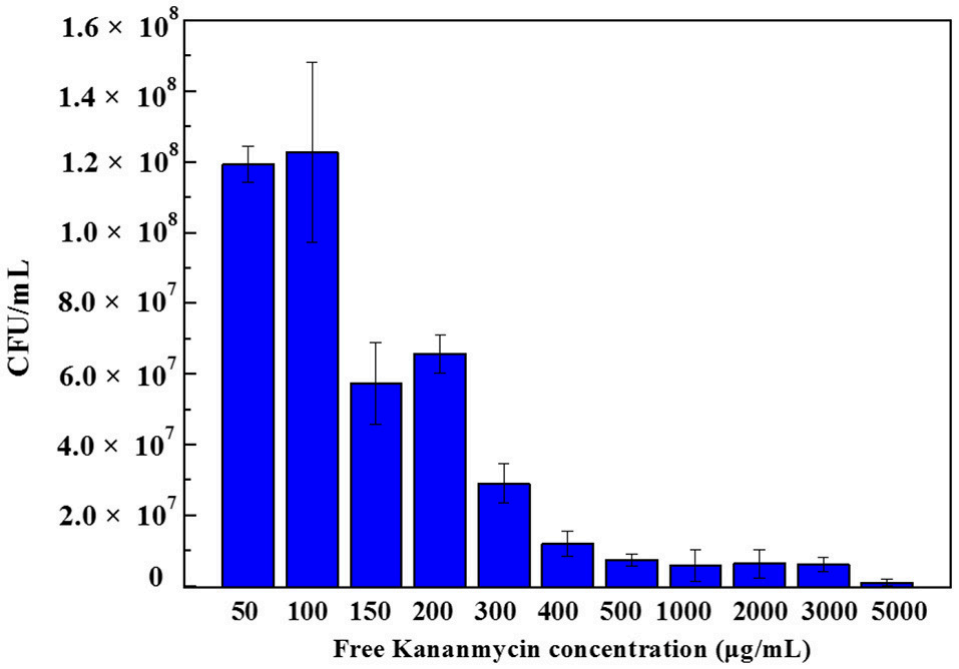
Colony forming units of *E. coli* bacteria with different concentration of free kanamycin on agar plates. Each experiment was repeated three times and error bars show the standard error of the experimental results.

**Figure 8:**
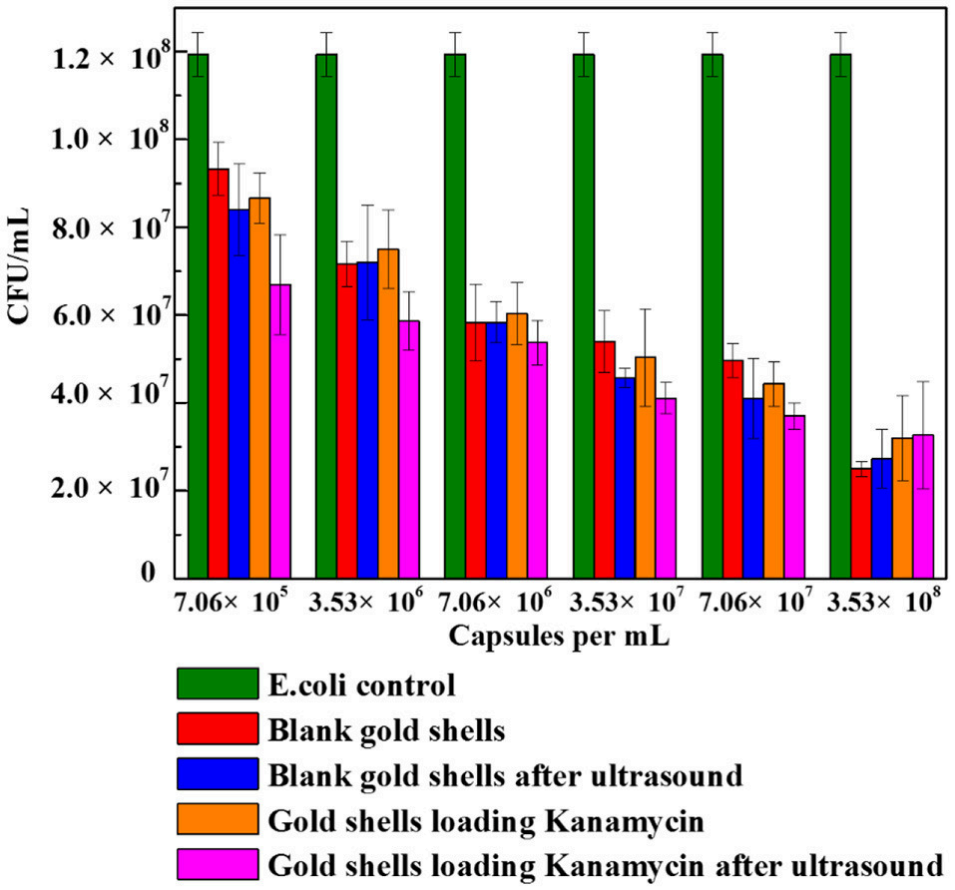
Cell viability of control *E. coli* and the ones with different numbers of gold coated colloidosomes. Each experiment was repeated three times and error bars show the standard error of the experimental results.

**Figure 9:**
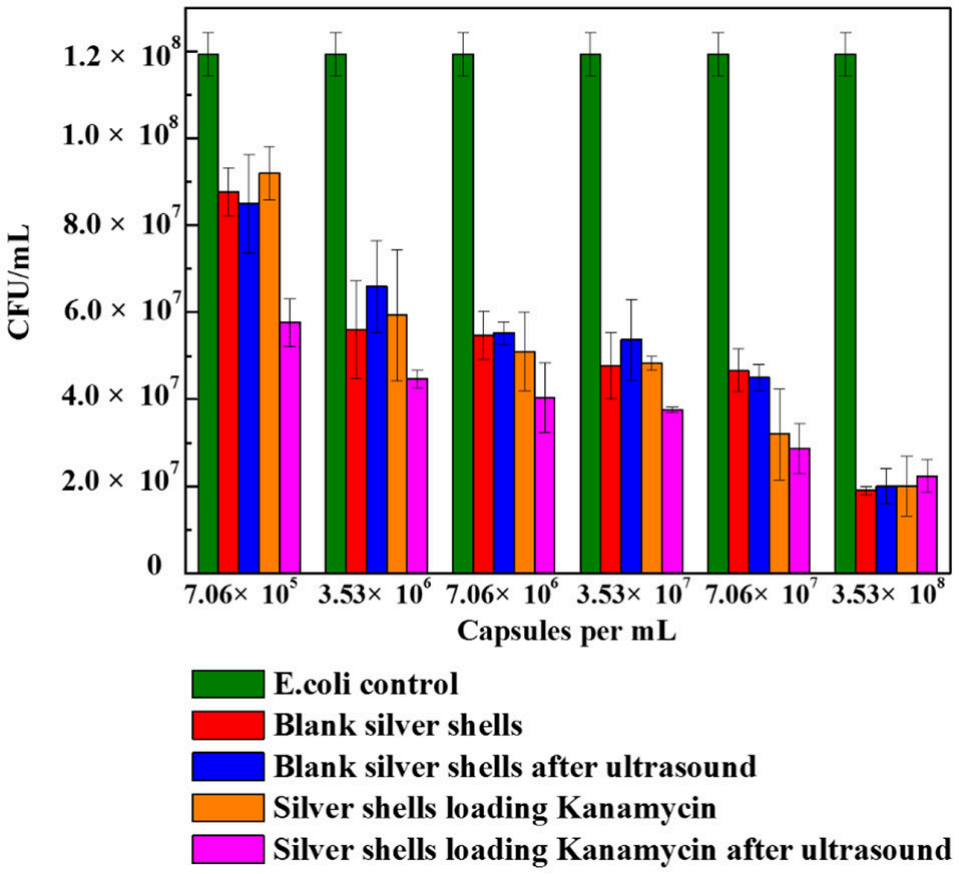
Cell viability of control *E. coli* and the ones with different numbers of silver coated colloidosomes. Each experiment was repeated three times and error bars show the standard error of the experimental results.

The application of the metal coated colloidosomes as small molecule drug carriers was investigated in a biological system. The CFU/mL data in Figures 7–9 are calculated by counting the average number of the diluted cell colonies. Each experiment was repeated three times and the standard error of the results is shown by the error bars.

#### Free kanamycin

Figure 7 shows the colony forming units results obtained after 24 h incubation of *E. coli* bacteria with different concentrations of free kanamycin. As can be seen, at a concentration of 50 and 100μg/mL kanamycin, the bacteria grew to 1.2 × 10^8^ CFU/mL. When the concentration increased to 150μg/mL, the numbers of CFU dropped to was 5.7 × 10^7^ CFU/mL.When the concentration increased to 200μg/mL, the result was increased slightly to 6.6 × 10^7^ CFU/mL, although within the error of the experiment. The *E. coli* strain being investigated contains a pET-24a(+) plasmid with a kanamycin resistant gene, which produces aminoglycoside modifying enzymes. Such strains survive the kanamycin treated media at low drug concentrations. Therefore, by adding certain amount of kanamycin, we can control that the cells being grown are the kanamycin resistant cells, and are not contaminated by any environmental bacteria. A low concentration of kanamycin shows a beneficial effect for cell growth. With an increasing the amount of kanamycin, the inhibition of cells increased. There is a complete inhibition for *E. coli* at 5000μg/mL kanamycin, when nearly all of the cells were killed.

#### Gold coated colloidosomes cell viability test

Figure 8 shows the cell viability results obtained after 24 h incubation of *E. coli* bacteria with different number concentrations of blank gold coated colloidosomes, blank gold coated colloidosomes after ultrasound, gold coated colloidosomes loading kanamycin, and gold coated colloidosomes loading kanamycin after ultrasound. In order to break the gold shells, ultrasound was applied for 480 s, before adding the resulting solution to the bacteria.

As seen in Figure 8, there was a CFU decrease in all of the gold shell treated samples compared to the blank *E. coli* culture with 50μg/mL kanamycin. This indicates the gold coated colloidosomes are toxic to the *E. coli* culture. The toxicity increases with increasing concentration of gold coated colloidosomes. With a concentration of 7.06 × 10^5^ capsules/mL the toxic effect from the colloidosomes after ultrasound results in a cell viability of approximately 6.7 × 10^7^ CFU/mL, a 25% drop from the case without ultrasound. Increasing the concentration of capsules, the toxicity becomes more obvious. When the number of the capsules reaches to 3.53 × 10^8^ capsules/mL, the encapsulation of kanamycin had minimal extra benefit, with the capsules themselves hindering the cell viability.

#### Silver coated colloidosomes cell viability test

Figure 9 shows the cell viability results obtained after 24 h incubation of *E. coli* with different amounts of blank silver coated colloidosomes, blank silver coated colloidosomes after ultrasound, silver coated colloidosomes loading kanamycin, and silver coated colloidosomes loading kanamycin after ultrasound. Before rupture, there is minimal toxicity difference between the empty and kanamycin containing capsules. After ultrasound, the kanamycin has a significant effect on cell viability (Tsao and Hall, 2017).

In order to break silver shells, the ultrasound was applied for 240 s before adding the resulting solution into the bacteria. The effect of the silver coated colloidosomes on cell viability shows a similar trend to the gold capsules, with the cell viability decreasing, compared with the *E. coli* control. The effect on cell viability of silver coated colloidosomes loading kanamycin after ultrasound was most dramatic because of the released antibiotic. For the lowest concentration of silver shells, the cell viability after kanamycin was released dropped by 34% compared with other capsules. Increasing the concentration of capsules, the toxic effect becomes enhanced.

Both the silver and gold shells colloidosomes are toxic to this bacterial system. With a low concentration of 7.06 × 10^5^ capsules/mL, the difference of toxicity between gold and silver colloidosomes is not significant. Increasing the concentration of capsules, the cell viability is slightly different. For example, at a concentration of 3.53 × 10^8^ capsules/mL, for gold shells after ultrasound, there are around 3 × 10^7^ CFU remaining, and for silver shell capsules after ultrasound around 2 × 10^7^ CFU remain. This slight difference is likely to be because the silver shells, especially silver shell fragments in nano-size as well as any silver ions are toxic to biological systems and are leading to death of bacteria. (Zhou et al., 2008; Mu et al., 2014).

## Conclusion

This paper has demonstrated a method for encapsulating an antibiotic kanamycin using gold or silver coated colloidosomes. The colloidosomes are impermeable and can be triggered using ultrasound. To investigate the application of the capsules in a biological system, *E. coli* bacteria were chosen as a model organism. After triggering the metal shells by ultrasound, the released antibiotic, the broken fragments, and the antibiotic loading on the capsule surface all kill *E. coli*. Both the silver and gold shells colloidosomes are toxic to this bacteria system. Compared with silver coated colloidosomes, gold ones can load a higher concentration of kanamycin.

## Author contributions

QS made the capsules and characterized them. QS and ZZ performed the cell viability experiments together. QS wrote the manuscript with assistance from ZZ, EH, and AR. EH and AR supervised the research.

### Conflict of interest statement

The authors declare that the research was conducted in the absence of any commercial or financial relationships that could be construed as a potential conflict of interest. The handling Editor and reviewer, SF, declared their involvement as co-editors in the Research Topic, and confirm the absence of any other collaboration.
